# Hematopoietic and leukemic stem cells homeostasis: the role of bone marrow niche

**DOI:** 10.37349/etat.2024.00262

**Published:** 2024-08-15

**Authors:** Shaimaa Khattab, Manal El Sorady, Ashraf El-Ghandour, Giuseppe Visani, Pier Paolo Piccaluga

**Affiliations:** Kumamoto University, Japan; ^1^Biobank of Research, IRCCS Azienda Ospedaliera-Universitaria di Bologna Policlinico di S. Orsola, 40138 Bologna, Italy; ^2^Department of Medical and Surgical Sciences, Bologna University School of Medicine, 40138 Bologna, Italy; ^3^Medical Research Institute, Hematology department, Alexandria University, Alexandria 21561, Egypt; ^4^Department of Internal Medicine, Faculty of Medicine, Alexandria University, Alexandria 5310002, Egypt; ^5^Hematology and Stem Cell Transplant Center, Azienda Ospedaliera Marche Nord, 61121 Pesaro, Italy

**Keywords:** Hematopoietic stem cell, acute myeloid leukemia, bone marrow niche, targeted therapy, drug resistance, precision medicine, immune system

## Abstract

The bone marrow microenvironment (BMM) has highly specialized anatomical characteristics that provide a sanctuary place for hematopoietic stem cells (HSCs) that allow appropriate proliferation, maintenance, and self-renewal capacity. Several cell types contribute to the constitution and function of the bone marrow niche. Interestingly, uncovering the secrets of BMM and its interaction with HSCs in health paved the road for research aiming at better understanding the concept of leukemic stem cells (LSCs) and their altered niche. In fact, they share many signals that are responsible for interactions between LSCs and the bone marrow niche, due to several biological similarities between LSCs and HSCs. On the other hand, LSCs differ from HSCs in their abnormal activation of important signaling pathways that regulate survival, proliferation, drug resistance, invasion, and spread. Targeting these altered niches can help in better treatment choices for hematological malignancies and bone marrow disorders in general and acute myeloid leukemia (AML) in particular. Moreover, targeting those niches may help in decreasing the emergence of drug resistance and lower the relapse rate. In this article, the authors reviewed the most recent literature on bone marrow niches and their relations with either normal HSCs and AML cells/LSC, by focusing on pathogenetic and therapeutic implications.

## Introduction

The bone marrow microenvironment (BMM) has highly specialized anatomical characteristics that provide a sanctuary place for hematopoietic stem cells (HSCs) that allow appropriate proliferation, maintenance, and self-renewal capacity [1, 2]. Two specialized microenvironmental niches have been described namely, the “osteoblastic (endosteal)” and the “vascular” niche. Direct cell-to-cell contacts and receptor-ligand interactions have long been considered the most predominant method of intercellular communication inside the BM niches [3].

Acute myeloid leukemia (AML) is an aggressive and heterogeneous form of acute leukemia that predominantly affects adults. It is marked by the abnormal proliferation of immature blood cells in the BM, which disrupts the production of normal blood cells [4]. Unfortunately, the current treatment for AML is suboptimal with a high relapse rate even after aggressive chemotherapy. Notably, leukemia cells can use the BM niche to evade cell death induced by chemotherapy and acquire drug resistance [5]. Moreover, there is growing evidence suggesting that leukemia cells have the ability to convert healthy BM niches into malignant ones, thereby promoting the survival and proliferation of leukemic stem cells (LSCs). A deeper comprehension of the biology of the AML niche is essential to develop more effective therapeutic options for enhancing patient outcomes [1, 6] (Table 1).

**Table 1:** Summary of different cell types and their role in the normal and leukemic HSC niches

**Cell type**	**Secreted or expressed factors (molecular pathways)**	**Healthy niche**	**AML niche**	**Altered molecular pathways**	
Non-hematopoietic cells					
Osteoblasts	CSF3 BMPRIA Osteopontin/CD44	Control HSC expansion and self-renewal Regulation of HSC homing, quiescence, and mobilization Control the generation of adaptive immune cells	AML decreases osteoblast production AML decrease OSP AML increases CD44 expression	Unchecked activation of the RANK/RANKL pathway	
Osteocytes	CSF3 (indirect way)	Regulation of osteoblast	**-**	**-**	
Nestin^+^ mesenchymal cells	CXCL12 SCF	Differentiation to osteoblast, chondrocytes and adipocytes Enhance HSC differentiation and maintenance of HSC Mediate sympathetic circadian signaling to HSC	MSCs increase CD271^+^ MSCs JAG1 MSCs decrease SCF Enhancement of BM homing of AML LSCs Supporting low proliferative but highly resistant LSCs	CXCL12/CXCR4	
CAR cells	CXCL12	Maintaining HSC Maintaining lymphoid progenitors	CXCR4 expression is increased by AML blast CXCL12 secretion is increased by CAR cells	CXCL12/CXCR4 Activation of JAK/STAT, PI3K/AKT, and MEK/ERK pathways through CXCL12/CXCR4	
Non-myelinating Schwann cells	TGFB (SMAD) signals	HSC quiescence	Disruption of nestin GFP^+^ cell quiescence	**-**	
SNS	Catecholamines	Controlling the cyclical circadian release of HSCs	SNS denervation enhances leukemic cell proliferation	Altered B2-adrenergic pathway	
Adipocytes	Adiponectin	Negative regulators of homeostatic	AML cells increase lipolysis AML cells increase FAO	GDF15 is highly expressed by LSCs Upregulation of *PPARG*, *FABP4*, and *BCL2* genes by LSCs	
Endothelial cells	Selectin E Selectin P VCAM1 Express Notch ligands	Doorkeeper Maintaining HSCs Increase HSC quiescence and self-renewal	Promote adhesion of LSCs Stimulates AML cells and proliferation Angiogenesis	Notch pathway CD44/Selectin E VLA4/VCAM1 DLL4 pathway	
Hematopoietic cells					
Osteoclast	RANKL pathway Proteolytic enzymes	Maintenance of the endosteal niche cavities HSC mobilization Osteoclast depletion is associated with an increase in EH	**-**		**-**
Macrophages	Oncostatin M Prostaglandin E2	HSCs mobilization HSCs retention in the endosteal niche Macrophages increase CXCL12/CXCR4 by nestin^+^ MSCs Promote Erythropoiesis	LAM helps in chemotherapeutic resistance		**-**
Lymphocytes	**-**	Regulation of hematopoietic stem cell growth T-regs play a crucial role in immune tolerance T-regs create a protective zone from the immune attack	AML dysregulates T-cells AML evades detection by NK cells Exhaustion of CD8^+^ T-cells Dysregulation in Th1/Th2 axis Increase in dysregulation in Th1/Th2 axis and its cytokines AML increase T-regs AML stimulates T-regs		TIM3/LGALS9 ICOSLG
Neutrophils	Indirect role mediated by macrophages	Clearance of aged neutrophils by macrophages regulates HSCs mobilization	**-**		**-**

HSC: hematopoietic stem cell; AML: acute myeloid leukemia; RANK: receptor activator of nuclear factor κB; RANKL: receptor activator of nuclear factor κB ligand; MSCs: mesenchymal stromal cells; BM: bone marrow; LSCs: leukemic stem cells; CAR: CXCL12-abundant reticular; TGFB: transforming growth factor-beta; GFP: green fluorescent protein; FAO: fatty acid β-oxidation; EH: extramedullary hematopoiesis; LAM: leukemia-associated macrophage; NK: natural killer; T-regs: regulatory T-cells; BMPRIA: bone morphogenetic protein receptor type IA; VCAM1: vascular adhesion molecule 1; SNS: sympathetic nervous system; VLA4: very late antigen 4; GDF15: growth differentiation factor 15; OSP: osteopontin. -: no data

## Sites of hematopoiesis

Hematopoiesis is the process of generation and replenishing the blood system with new blood cells that occur throughout embryonic and adulthood. In embryonic life, hematopoiesis occurs in the yolk sac and the aorta-gonad-mesonephros (AGM) region then it shifts to the placenta fetal liver, and spleen, and then to the BM, which is the location for the HSCs in adults [7]. On the contrary, the yolk sac contribution to the adult system cannot be excluded [8]. Recent evidence pointed to the existence of self-renewing tissue-resident macrophage subsets, most of them, originate from yolk sac hematopoiesis, especially microglia of the brain [9, 10]. Hematopoiesis has two waves: the primitive wave and the definitive wave [11]. The primitive wave, which occurs during early embryonic life, gives rise to erythrocytes and macrophages from erythroid progenitors. The main objective of the primitive wave is to rapidly produce large amounts of red blood cells to help tissue oxygenation for rapidly growing embryos. Definitive hematopoiesis occurs later, in which HSCs produce all hemopoietic elements [12].

Hematopoiesis has to be controlled in a precise and rapid manner to meet the varying demands of homeostatic and stressful conditions [2]. BM is the final microenvironment niche for HSCs in adulthood which begins to host HSCs after the fetal liver ceases, it supports HSCs self-renewal, maintenance, and differentiation [13]. The majority of blood cells in adult humans are produced in the axial skeleton. This includes the skull, sternum, ribs, vertebrae, and iliac bones. The conversion to fat marrow is a gradual, steady, progressive process occurring at different rates in different bones and at different rates within the same bone [14].

Extramedullary hematopoiesis (EH) is the occurrence of hematopoiesis in organs other than BM. Physiological EH is the one that occurs in the fetal liver and spleen during embryogenesis. Pregnancy also can result in physiological EH. It has been reported that pregnant women are enriched with CD71^+^ erythroid cells [15]. The increased presence of transforming growth factor-beta (TGFB) during pregnancy facilitated the differentiation of CD34^+^ hematopoietic stem and progenitor cells into CD71^+^ erythroid cells [16]. Pathological hematopoiesis occurs during infection to augment the immune response through the overproduction of phagocytic cells and antigen-presenting cells (APCs). Additionally, EH occurs as a result of pathological myelopoiesis such as myelofibrosis as the marrow niche becomes inhabitable [17]. Surprisingly, the process by which LSCs migrate into extramedullary tissue is similar, or quite comparable to, that of regular stem cells. In fact, studies have shown that in mice xenotransplantation models, myeloid leukemia cells utilize traditional adhesion receptors to bind to endothelium and aid their migration into organs [18]. Of note, LSCs invade extramedullary organs after transmigrating local endothelial cell (EC) barriers. Transmigration is dependent on several adhesion molecules (integrins, selectins), and is enhanced by various proteases, cytokines, and histamine. After transmigration into extramedullary organs, LSCs invade local tissue sites. Invasion is facilitated by several surface molecules such as CD44 [19, 20], and is promoted by chemotactic factors (chemokines), proteases, and cytokines. Additionally, after LSC migration, there is often an increase in stem cell proliferation and the emergence of specific groups of self-renewing cells that propagate the disease. Additionally, there is a suggestion that leukemic cells may have the ability to create their own stem cell environments, which could help them resist drugs and promote self-renewal in LSCs [21–23].

## Hematopoietic stem cell and BM niche

**Figure 1:**
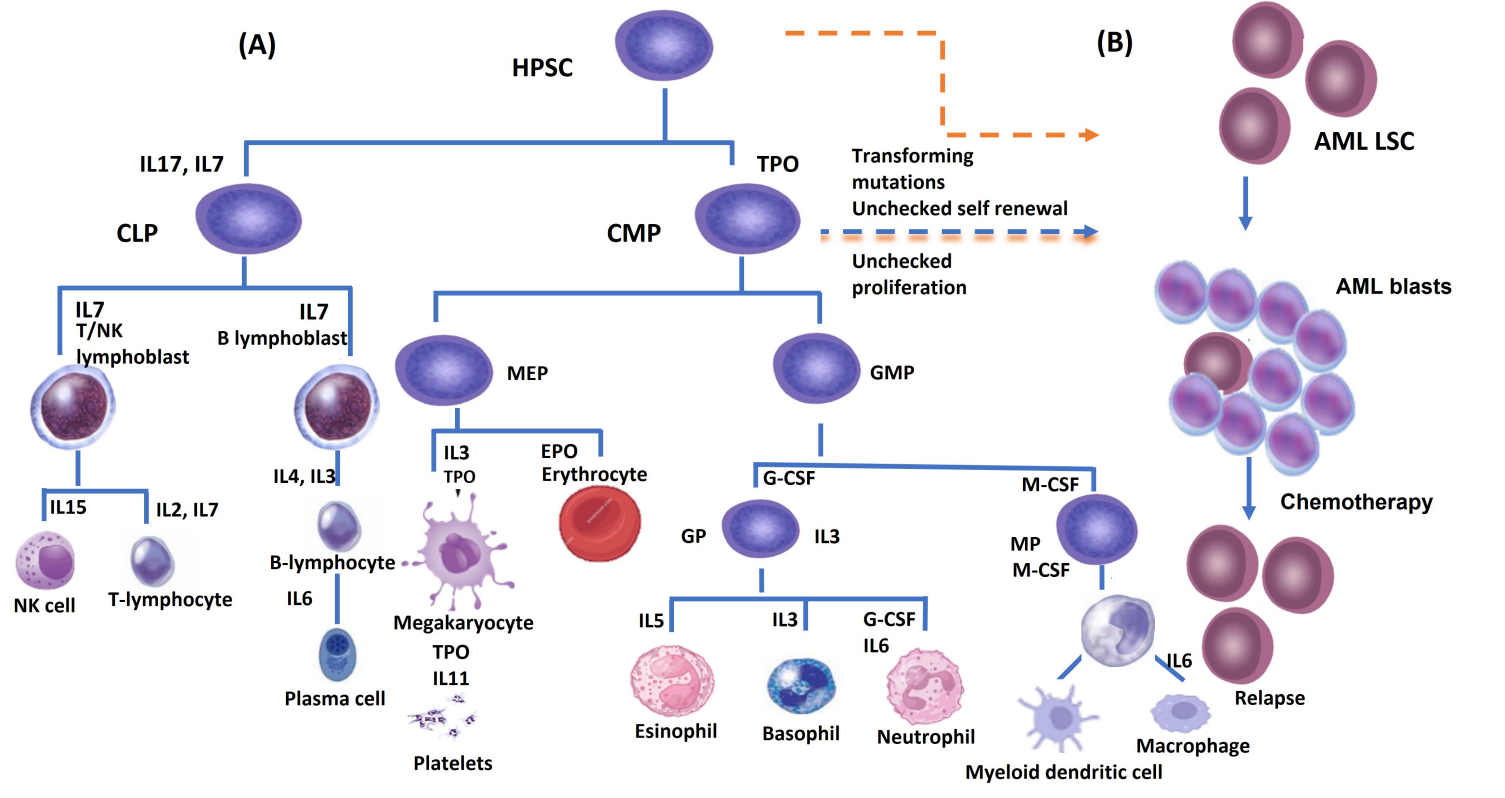
(A) Hematopoietic stem cells, which can undergo either self-renewal or hierarchical differentiation into lineage-committed progenitors that will give rise to various mature blood cells. Cytokines and their receptors are necessary for the progenitors to pass through the different maturation steps; (B) leukemic stem cells (LSCs) originate from transforming mutations in hematopoietic stem cells (HSC) and/or common myeloid progenitor (CMP). These mutations impact the self-renewal ability of LSCs and result in changes in survival signaling pathways. Chemotherapy can clear the acute myeloid leukemia (AML) blast cells population while the LSCs survive due to their quiescent state, and affect the rate of relapse. CLP: common lymphoid progenitor; GMP: granulocyte macrophage progenitor; GP: granulocyte progenitor; T/NK cell: T and natural killer cell; MP: macrophage progenitor; TPO: thrombopoietin; SCF: stem cell factor; IL: interleukin; G-CSF: granulocyte colony-stimulating factor; M-CSF: monocyte colony-stimulating factor; HPSC: human pluripotent stem cell; MEP: megakaryocyte-erythroid progenitor; EPO: erythropoietin

HSCs are scarce in number in BM [24] and have the substantial properties of the ability to self-renewal, and multipotency that sustain multilineage hematopoiesis so continuous proliferation and differentiation into lineage-specific, unipotent, progenitors responsible for the production of each major blood lineage [25] (Figure 1). There are two types of HSCs, the long term-hematopoietic stem cells (LT-HSCs) (Lin^−^CD34^−^CD38^−^CD93^hi^) [26] have lifelong self-renewing potential, whereas the short-term HSCs (ST-HSC) (Lin^−^CD34^+^CD38^−^CD45RA^−^CD49f^+^CD90^+^) [27] show more restricted self-renewing capacity [12, 28, 29]. Schofield [30] proposed that for hematopoiesis to take place, a particular microenvironment in the BM must provide the necessary autocrine, endocrine, and paracrine signals that enable HSCs to renew themselves and differentiate into all types of blood cells. Additionally, it is required to enable direct cell-to-cell interactions [30]. The BMM is composed of micro-niches, those niches stimulate and regulate several responses in HSCs such as homing, mobilization, quiescence, self-renewal, or lineage commitment [28].

**Figure 2:**
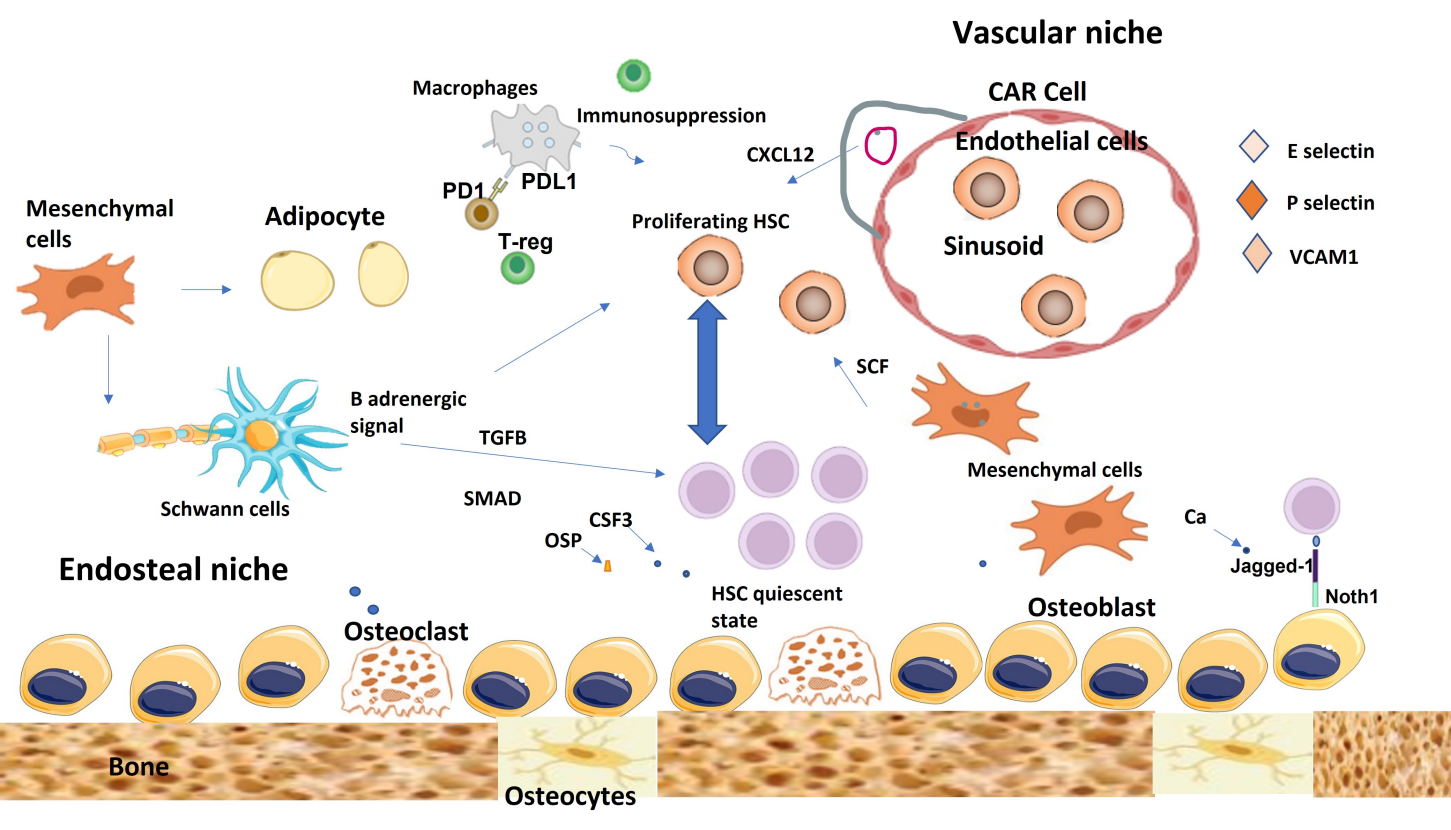
The endosteal niche and vascular niche of the healthy bone marrow. The endosteal niche is mainly involved in maintaining hematopoietic stem cell (HSC) quiescence, while the vascular niche promotes, differentiation, proliferation, and mobilization of HSCs. Mesenchymal cells can differentiate into several types of cells including adipocytes and osteoblasts. CAR cells maintain HSC through the secretion of CXCL12. Non-myelinating Schwann cells help in HSC quiescence through TGFB and SMAD signals. Immune cells help to create immunosuppressive microenvironment to protect HSC from immune attacks. TGFB: transforming growth factor-beta; T-reg: regulatory T-cell; CAR: CXCL12-abundant reticular; PD1: programmed death 1; PDL1: programmed death ligand 1; OSP: osteopontin; SCF: stem cell factor

There are two definitive types of niches, endosteal niches and perivascular niches [31]. They are composed of several different cells; however, there is not a definitive consensus on whether they might contribute to both or either one only (Figure 2).

### Osteolineage cells

In normal BM, HSCs reside very near the endosteal bone surface [32]. It was found that HSCs that reside in this type of niche have a highly proliferative capacity and long-term self-renewal properties [33]. The cellular component of the endosteal niche typically involves osteoblasts (the main cells supporting myelopoiesis), osteocytes, osteoclasts and Schwann cells, and regulatory T-cells (T-regs).

The osteoblasts are the primary cells that form a connection between bone and the BM in the endosteal niche. Osteoblasts can be divided into two types: oval-shaped osteoblasts and spindle-shaped *N*-cadherin + osteoblasts. Both play an essential role in the regulation of hematopoiesis [34]. Additionally, they control HSC expansion and self-renewal in vivo through colony-stimulating factor 3 (CSF3) [35]. The communication between osteoblasts and HSCs in the endosteal niche can be facilitated through various mechanisms as osteoblasts produce several cytokines and growth factors that regulate HSC homing, quiescence, and mobilization.

One study has shown that an increased number of osteolineage cells, correlated with an increased number of HSCs through the action of activated parathyroid hormone receptor [35], and another one described the effect of the bone morphogenetic protein receptor type IA (BMPRIA) depletion [34]. It has been proven that osteolineages cells are essential players that control the generation of cellular elements of adaptive immune response like B and T lymphocytes [36–38].

Osteoclasts play an essential role in bone remodeling by creating resorption pits (Howship’s lacunae), they are multinucleated cells found in bone. Osteoclast progenitors originate from hematopoietic cells in BM and blood vessels that supply bone [39]. It is believed that osteoclasts are implicated in the maintenance of the endosteal niche cavities through the receptor activator of the nuclear factor kappa B ligand (RANKL) [40]. Osteoclasts have been involved in HSC mobilization as a response to stressful conditions and therapeutic agents such as CSF3 [41] through the generation of some proteolytic enzymes that cleave factors involved in the HSC niche [42]. Additionally, osteoclast depletion is associated with impairment in normal hematopoiesis and an increase in EH [43].

### Mesenchymal stromal cells (MSCs)

Mesenchymal stromal cells (MSCs) also called stromal cells are located near BM blood vessels and they are capable of self-renewal and differentiation into osteolineage cells, chondrocytes, adipocytes, and myofibres [44]. The progenitor of BM MSCs has recently been identified as skeletal stem cells (SSC) based on their differentiation capabilities [45]. MSCs are distinct from HSCs, they do not express CD45, CD34, CD41, CD14, and T- or B-cell markers [46].

MSCs expressing the intermediate filament protein nestin (nestin^+^ MSCs) enhance HSC differentiation by constitutive secretion of several hematopoietic cytokines and in response to interleukin 1 (IL1) [47]. Because of their substantial role in hematopoiesis, MSCs have been investigated in allogeneic HSC transplants [48] as they proved their ability to enhance engraftment in animal models [49]. On the contrary, the ablation of nestin⁺ MSCs in BM transplantation recipients also reduces hematopoietic stem and progenitor cells (HSPCs) homing into the BM by 90%, and they were found to be localized with Lin^−^CD48^−^CD150⁺ HSCs, pointing at their role as niche cells [41]. Additionally, the cultivated MSCs express a variety of bioactive compounds with anti-apoptotic, immunomodulatory, angiogenic, anti-scarring, and chemoattractant qualities. These findings support the use of MSCs as a potential resource to establish local regeneration environments in vivo [50].

### CXCL12-abundant reticular (CAR) cells

CXCL12-abundant reticular (CAR) cells are a small number of reticular cells that are considered a subpopulation of mesenchymal cells with long processes, expressing high amounts of CXCL12 and its receptor (CXCR4) [51] play a crucial role in maintaining the pool of HSCs and their lymphoid progenitors. In fact, this proves that CAR cells are essential cellular niches for HSCs. In vivo, ablation of CAR cells results in reduced HSC cycling, increased expression of myeloid commitment markers such as PIRAT1 and CSF1R, and major loss of adipogenic and osteogenic capacity [52].

### Bone marrow endothelial cells (BMECs)

BMECs typically line the BM sinusoidal system and also make cell-to-cell contact with other cell types within the BM environment, owing to their anatomical characteristics they act as a doorkeeper and a major barrier that regulates cellular and chemical movement between the BM and the blood circulation [53]. Additionally, the BM endothelium can also influence hematopoiesis through cytokines secretion, even without cell-to-cell interaction [54].

ECs play a direct and indirect role in maintaining HSCs through an expression of the Notch ligands JAG1, JAG2, DLL1, and DLL4. They activate the Notch pathway, which helps in the self-renewal of LT-HSCs in vitro and the rebuilding of the LT-HSC pool in vivo after myeloablation [55]. Additionally, it is proven that ECs secrete various cytokines and express multiple adhesion molecules such as selectin E, selectin P, and vascular adhesion molecule 1 (VCAM1) [56], it is worth mentioning that selectin E, expressed exclusively by ECs, promoted HSC proliferation, as demonstrated by the increased HSC quiescence and self-renewal [57].

### Adipocytes

It has been proposed that fat from different tissues differs from BM fat. Bone marrow adipose tissue (BMAT) seems to be smaller in size, distributed throughout the BM, rather than having been arranged into lobules. The number of yellow marrow is increased by age and it is accompanied by a decrease in HSCs in return, studies on mice treated with adipogenic inhibitors showed an increase in HSCs engraftment, thus adipocytes act as a negative control of hematopoiesis [58]. It is claimed that the BMAT can secrete substances such as cytokines and hormones to contact with other cells in the BM environment. Additionally, BMAT exhibits different genes compared to compared to subcutaneous adipocytes [59]. Furthermore, BM adipocytes can secret leptin which may help in myelopoiesis [60].

### Neural cells and the nervous system

It has been determined that sensory and autonomic innervation of the BM helps in the process of the HSC niche regulation [61]. The sympathetic nervous system (SNS) plays a substantial role in controlling the cyclical circadian release of HSCs through the effect of the secreted catecholamines on (CXCL12) by MSCs through the B3-adrenergic receptor [61]. Moreover, it was proposed that non-myelinating Schwann cells may help in HSC quiescence and hibernation through activation of TGFB (SMAD) signaling [62].

### Megakaryocytes

Megakaryocytes are a huge in size but scarce population of BM cells with a lobulated nucleus, they are highly specialized precursor cells that are responsible for the production and release of platelets into the circulation. Recently, several investigations have revealed that megakaryocytes play new roles in controlling the physiology and homeostasis of the BM. Recently, it has been discovered that megakaryocytes have new roles in regulating the BM’s physiology and homeostasis [63].

It has been suggested that there is a definite interaction between megakaryocytes, HSCs, and osteolineage niche within the BM, as megakaryocytes increase osteoblast proliferation after total body irradiation [64]. After BM transplantation, osteoblasts proliferate rapidly in response to mesenchymal growth factors released by megakaryocytes, such as platelet-derived growth factors. This process helps with HSC engraftment and hematopoietic reconstitution [65].

Megakaryocytes secrete various cytokines [e.g., thrombopoietin, TGFB, fibroblast growth factor 1 (FGF1), and CXCL4] through them they can help in regulating HSCs. Thrombopoietin is responsible for the migration of megakaryocytes to the endosteum and its administration to megakaryocyte-depleted mice helps restore HSCs’ quiescence state [66]. TGFB secreted by megakaryocytes maintains HSCs quiescence in the physiological state, on the other hand, under stressful conditions FGF1 promotes HSC expansion [67]. Additionally, CXCL4 secreted by the megakaryocytic pool exerts a negative control effect on HSC proliferation and decreases engraftment [68].

### The immune cellular system and BM niche

The BM creates a specialized immune-privileged environment that protects HSCs from any attack from the immune system by suppressing the immunological responses, which even allows foreign allografts to survive for longer periods. This protection is achieved through a complex interplay of various systems within the body [69].

#### Lymphocytes

Lymphocytes are essential for cellular and humoral immunity, they can also influence hematopoiesis, through direct cellular contact with the HSCs. It has been proved that natural killer (NK) cells or large granular lymphocyte (LGL) are implicated directly or via humoral factors in the homeostasis and regulation of HSC growth and differentiation and it has a negative impact on HSC differentiation [70]. CD4^+^ T-cells play a crucial role in BM hematopoiesis, CD4^+^ T-cells depletion in mice resulted in the production of myeloid progenitors with impaired differentiation capacity [71].

T-regs are a subpopulation of T-lymphocytes with a characteristic immunophenotype (CD3^+^CD4^+^CD25^+^CD127^−^) along with intracellular (FoxP3) expression [72]. T-regs play a crucial role in immune tolerance and immune homeostasis. They downregulate immune responses in a variety of autoimmune and inflammatory conditions [73]. At the HSCs level, T-regs can help in creating a protective zone from the immune attack as it was found in the mice models that HSCs were lost after T-regs removal [69].

Programmed death 1 (PD1) is expressed on pro-B and activated T-cells and it has two ligands, programmed death ligand 1 (PDL1) and PDL2. PDL1 is expressed on almost all types of lymphoid cells also it is found on the surface of other hematopoietic cells and abundant in leukemia cells. On the contrary, PDL2 is expressed primarily in macrophages and dendritic cells. Normally, the PD1 system acts through a feedback loop that inhibits the T-cell receptor (TCR) signals, T-cell activation, and cytokine release, this acts as a critical mechanism to prevent autoimmune attacks against self-cells [74, 75].

#### Macrophage

CD169^+^ macrophages play a crucial role in HSCs mobilization through signaling the MSCs and they promote HSCs retention in the endosteal niche. It was found that they are depleted during therapeutic usage of CSF3 to induce HSCs mobilization, and their loss facilitates HSCs mobilization to the peripheral circulation [76]. CD169^+^ macrophages enhance HSC retention in the BM niche through the secretion of a soluble factor called oncostatin M that regulates the expression of CXCL12 by nestin^+^ MSCs [77].

Recently a rare type of macrophages that express high levels of α-smooth muscle actin and cyclooxygenase 2 can produce prostaglandin E2, which increases CXCL12 expression in nestin^+^ MSCs and CXCR4 expression on HSCs [78]. Additionally, macrophages can regulate HSC mobilization from the BM to the peripheral blood (PB) circulation after phagocytosis of aged neutrophils [79]. It also serves as a niche cell that promotes erythropoiesis through unknown pathways [80].

#### Neutrophils

Neutrophils are one of the most numerous cells in circulation as they act as the first line of defense against invading microbes. They have a short circulating half-life as they migrate to the tissue in need. Their anti-microbial activity can be bitoxic as they can pathologically attack self-tissue if they are kept unchecked [81]. Neutrophils normally have serine protease activity which acts as a proteolytic enzyme that cleaves various cytokines and receptors crucial for HSC retention in vitro, including CXCL12, CXCR4, VCAM1, KIT, and stem cell factor (SCF) [24]. The apoptosis of the neutrophils by macrophage acts as a modulator of neutrophilic effect on the HSCs niche as it serves as a signal for HSC mobilization as mentioned before [79].

### Single-cell analysis of niche heterogeneity

The genomics revolution has enabled entire tissues, such as the hematopoietic system, to be interrogated comprehensively at single-cell resolution, revealing a valuable degree of functional and phenotypic variation within cell compartments presumed previously to be homogeneous. This heterogeneity reveals how cells decide their fate and differ in their roles in maintaining the hematopoietic niche. For instance, single-cell RNA sequencing (scRNA-seq) paves the road to identify, classify, and discover new or rare cell types and subtypes from different human organs and tissues, giving more profound information about health and disease in many fields [82]. Moreover, it allows the identification of thousands of genes in each cell, providing an unbiased characterization of their transcriptional diversity even within phenotypically homogenous populations [83].

Several landmark studies have illustrated the molecular complexity of the murine BM microenvironment based on scRNA-seq [2, 84]. In stressful conditions, some transcriptional remodeling of niche elements occurs like adipocytic skewing of perivascular cells, and downregulation of vascular Notch delta-like ligands (encoded by *DLL1* and *DLL4*) [2]. Baccin et al. [85] performed scRNA-seq on 7,497 cells, arranged into 32 clusters corresponding to distinct cell types or stages of differentiation. They also revealed two different subsets of CAR cells: a more abundant Adipo-CAR cell population expressing adipocyte-related genes located adjacent to sinusoids and a less abundant Osteo-CAR cell population (enriched in osteolineage-related genes) located in endosteal BM regions or associated with arteriole-like vessels.

Li et al. [86] used scRNA-seq to investigate the human non-hematopoietic BME aiming to identify potential novel marrow stromal subsets and cellular hierarchies as well as to establish functional relationships between stromal and hematopoietic elements. They identified six transcriptionally and functionally different stromal cell populations and demonstrated that certain types of MSCs have the highest colony-forming units-fibroblast (CFU-F) potential and trilineage differentiation capacity with astonishing capability of interaction with all Hematopoietic cells through CXCL12 pathways. Moreover, Severe et al. [87] were able to identify 28 distinct subsets of stromal cells in BM under steady-state conditions by using single-cell mass cytometry, although only half of them expressed hematopoiesis-relevant cytokines. They emphasized that the CD73^+^ stromal subset is likely to promote HSPC engraftment and acute hematopoietic recovery following radiation conditioning.

## LSCs niche in AML

The concept of cancer stem cell model was first described in AML [88, 89]. Cancer stem cell hypothesis proposes that cancer is propagated by a subpopulation of cells with stem cell properties, namely the ability to proliferate while balancing self-renewal with differentiation [90]. The leukemic clone is sustained by a scarce population of LSCs that have acquired a dramatic increase in their ability to self-renew [91]. Those rare AML cells characterized by a HSC CD34^+^CD38^–^ phenotype were believed capable of generating leukemia in immunocompromised mice [88, 89]. The clinical relevance of identifying LSC in human AML has remained controversial, as LSCs are extremely heterogeneous phenotypically and genetically [92, 93]. For instance, patients whose leukemic cells initiated leukemia in xenografts had significantly shorter overall survival (OS) than patients with leukemia cells that did not engraft [94]. Moreover, high CD34^+^CD38^−^ LSC burden at the time of diagnosis had also been associated with high minimal residual disease after chemotherapy treatment and poor OS [95]. Additionally, multivariate analysis showed aldehyde dehydrogenase (ALDH)^bright^ frequency, a LSC marker, to be the strongest prognostic factor for OS compared to other risk factors such as age, genetic aberrations, blast count, and frequency of CD34^+^ cells [96]. It is well known that some patients who test negative for minimal residual disease (MRD) experience a recurrence in every immunophenotype and/or molecular MRD study. One of the possible biological explanations of this discrepancy is the fact that not only the number of leukemic blast cells, reflecting MRD, that defines the risk of relapse but also the number of LSC present within this blast cell population [97]. Moreover, when the number of CD34^+^/CD38^−^ LSC after therapy was identified, LSC load was an independent predictive factor for patient survival [98]. Since normal CD34^+^/CD38^−^ cells possess similar features as LSC and the design of new therapies requires the specific eradication and monitoring of CD34^+^/CD38^−^ LSC it is mandatory to specifically discriminate LSC-containing fractions from HSC using more cell-surface markers [97, 99].

Due to several biological similarities between LSCs and HSCs, they share many signals that are responsible for interactions between LSCs and BM niches. However, LSCs differ from HSCs in their abnormal activation of important signaling pathways that regulate survival, proliferation, drug resistance, invasion, and spread [100]. Studies using xenograft and syngeneic mice models have shown that leukemic cells are in competition with healthy HSCs for the HSC niche. This competition interferes with the normal interactions that HSCs have with their surroundings, which causes HSCs to be displaced from their niche and results in impaired hematopoiesis [101].

**Figure 3:**
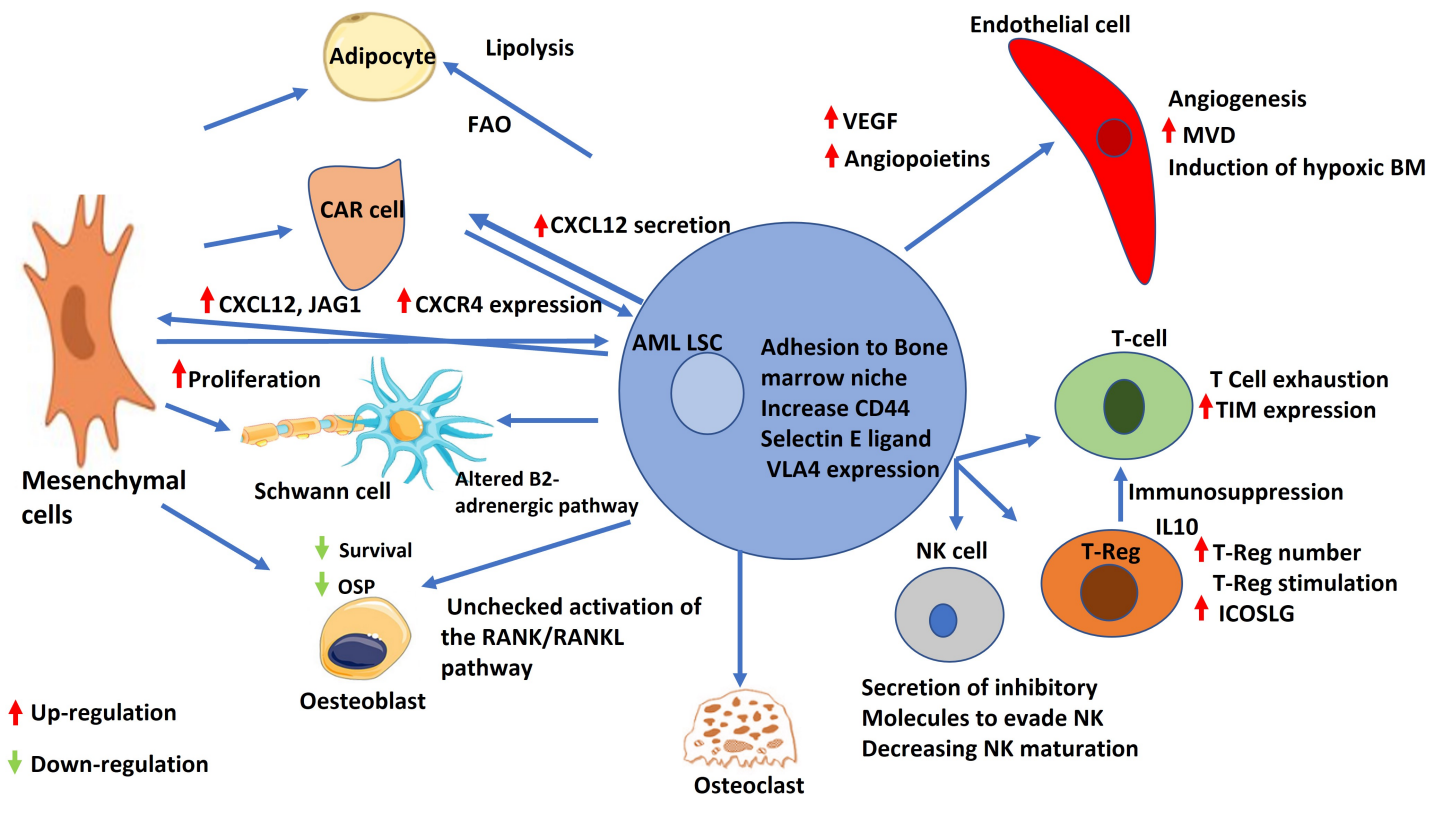
AML leukemic stem cell and its interaction with bone marrow niche cells. Schematic representation of the interactions between AML LSC and different components of the bone marrow niche. AML LSCs increase secretion of VEGF and angiopoietins. AML: acute myeloid leukemia; LSC: leukemic stem cells; FAO: fatty acid β-oxidation; CAR: CXCL12-abundant reticular; OSP: osteopontin; RANK: receptor activator of nuclear factor κB; RANKL: receptor activator of nuclear factor κB ligand; VEGF: vascular endothelial growth factor; VLA4: very late antigen 4; NK: natural killer; MVD: microvessel density; BM: bone marrow; TIM: T-cell immunoglobulin; IL10: interleukin 10

It has been pointed out that the creation of LSC-educated BMM is essential in the pathogenesis of AML (Figure 3). Several molecular mechanisms are involved such as angiogenesis, BMAT remodeling, adhesion factors, neural signals, hypoxia alteration [102], and exosomes [23]. Unraveling the secrets of BMM and its interaction with HSCs in health paved the road for research aiming at better understanding the concept of LSCs and their alter niche. Targeting these altered niches can help in better treatment choices for hematological malignancies and BM disorders in general (Table 2). Moreover, targeting those niches may help in decreasing the emergence of drug resistance and lower the relapse rate.

**Table 2:** Selected therapeutic targets on AML niche

**Mechanism**	**Targets**	**Cognate ligand in BM niche**	**BM niche**	**Drug**	**Clinical trials reference**
Adhesion of leukemic stem cell to bone marrow niche	CD44	Hyaluronan, osteopontin	Endosteal niche	Polymeric nanoparticle-mediated silencing of CD44	N/R
	Selectin E	Selectin E ligand	Vascular niche	Uproleselan	[108, 109]
	VLA4	VCAM1	Vascular niche	AS101	N/R
CXCL12/CXCR4 axis	-	CXCR4	Vascular niche	Ulocuplumab	[108, 109]
				Plerixafor	[119, 120]
				CXCR4 antagonist LY2510924	[121]
Angiogenesis	-	VEGF	Vascular niche	Aflibercept	N/R
				Bevacizumab	[136]
		Angiopoietins	Vascular niche	Trebananib	[139]
Bone remodeling signaling pathway	-	Proteasome inhibitors	Vascular niche	Bortezomib	[143]
				Carfilzomib	[144]
				Ixazomib	[145]
		Receptor tyrosine kinase inhibitors	Vascular niche	Cabozantinib	[146]
Bone marrow adipose tissue remodeling	-	FAO inhibitors	Vascular niche	Avocatin B	N/R
Tumor immune evasion	-	PD1 inhibitor	-	Nivolumab	[170]
				Pembrolizumab	[171, 172]
		Anti-CTLA4	-	Ipilimumab	[167]

-: no data. N/R: no reference; VLA4: very late antigen 4; VCAM1: vascular adhesion molecule 1; VEGF: vascular endothelial growth factor; FAO: fatty acid β-oxidation; PD1: programmed death 1; CTLA4: cytotoxic T-lymphocyte associated protein 4

### Adhesion of LSC to BM niche

Adhesion of LSCs to the BM niche is a crucial process in AML pathogenesis and progression. Establishing their roles and how they interplay can be a major therapeutic target in AML management. CD44 is a cell-surface glycoprotein that attaches to the extracellular matrix proteins hyaluronan, osteopontin, and selectin E. One study revealed that in comparison to normal HSCs, the expression of CD44 variant exons is more prevalent in AML cells [103] also some studies showed that high levels of CD44 molecules were essential for AML relapse in mouse models of AML [104]. Moreover, reducing surface levels of CD44 in AML cells by polymeric nanoparticles can induce apoptosis and reduce the adhesion of AML cells to mesenchymal stem cells in human BM [105]. Understanding the biology and the pathological impact of expression of CD44 in the maintenance of LSCs niche can be a potential therapeutic target for AML through the administration of anti-CD44 antibody-like in mice infected with human AML cells [103].

Selectin E, another adhesion molecule located in the BM vascular niche promotes the proliferation of normal HSC, one study, by using flowcytometry demonstrated that AML blast cells express selectin E ligands. Selectin E upregulates both the WNT and sonic hedgehog pathways in AML resulting in promoting AML blast cells survival so by targeting those molecules through the addition of GMI-1271 (uproleselan) to the AML initial therapy protocol a better outcome may be achieved [106–109].

Very late antigen 4 (VLA4), composed of α4 and β1, binds to both the CS1 domain of fibronectin (Fn) and VCAM1 and has a critical role in HSCs retaining in the BM niche. VLA4 is highly expressed AML blasts. Chemotherapy drug resistance that results from disturbance in adhesion is caused by either the activation of survival pathways or the suppression of apoptotic processes. Some examples of signal transduction pathways that may be involved include PI3KCA/AKT/BCL2, PI3K/ILK/AKT, and WNT/GSK3B/NF-κB. AS101 is an anti-VLA4 inhibitor in clinical research, it inhibits VLA4 by binding Fn and suppressing PI3K/AKT/BCL2 signaling leading to increased chemo-sensitivity in mouse xenograft of AML [110].

### Cytokines and chemokines

Cytokine signaling is crucial in maintaining BM niche homeostasis by precisely regulating cross-talks between the different cellular components. Not surprisingly, the malignant BMM mediates LSCs attachment to their niches by up-regulation of various cytokines and chemokines.

CXCL12 is one of the most crucial chemokines secreted by the MSC, in particular, CAR cells. CXCL12 is secreted in a large amount in AML. The interaction between stromal and leukemic cells is mediated by the interaction between CXCL12 and its CXCR4 receptor [111]. Notably, CD34^+^ AML leukemic blast cells have a huge amount of CXCR4 receptors on their surfaces [112] and migrate in response to CXCL12 [113]. The CXCL12/CXCR4 axis is now considered a crucial player in LSCs survivability and proliferation through the activation of proliferative and pro-survival pathways like the JAK/STAT, PI3K/AKT, and MEK/ERK pathways [112].

Several factors regulate CXCL12/CXCR4 axis in AML such as chemotherapy-induced stress and *FTL3* gene mutation status [114, 115]. The expression of CXCR4 on AML blast cells could be correlated with the adhesive and migratory potential of the leukemic cells within the BM niches [116]. Moreover, the prognostic significance of the CXCL12/CXCR4 axis in leukemia has been studied to develop risk-stratified treatment approaches. Elevated CXCR4 expression in LSCs has been found to correlate with a significantly poor outcome and high recurrence rate. Additionally, CXCR4/CXCL12 can be considered an indirect mechanism that plays an important role in drug resistance. A variety of inhibitors, peptides, and antibodies have been developed to efficiently destroy, and induce apoptosis in CXCR4-expressing cancerous cells [117]. Due to these characteristics, CXCR4 inhibitors are gaining more interest as a monotherapy or as part of an ablative chemotherapeutic regimen in the context of hematopoietic cell transplantation (HCT) [118]. For example, the tolerability and safety of plerixafor have been demonstrated in patients with AML undergoing allogeneic hematopoietic stem cell transplantation (HSCT) [119], and in phase 1 trials for patients with relapsed/refractory acute leukemia [120]. Additionally, LY2510924, another potent CXCR4 peptide antagonist, led to a phase 1 study that demonstrated safety and yielded a response signal in patients with relapsed/refractory AML in combination with Idarubicin, and cytarabine [121]. Furthermore, ulocuplumab is a human IgG4 monoclonal anti-CXCR4 antibody that has therapeutic potential for several hematologic malignancies including AML [122], clinical studies have demonstrated the ability of ulocuplumab to mobilise leukemic cells into the circulation, rendering them more susceptible to chemotherapy-induced toxicity in patients with AML [123, 124].

### Angiogenesis

The word “angiogenesis” comes from the Greek words “angeio” meaning blood vessel and “genesis” meaning creation. It refers to the process of forming new blood vessels. Angiogenesis enhances the growth of cancerous cells by continuously supplying them with nutrition, oxygen, and growth factors from the surrounding milieu. Various studies suggest that angiogenic factors and/or vascularization in solid tumors can be a crucial indicator for cancer treatments and have prognostic significance [125]. However, the significance of vascularization in “liquid tumors” or leukemias, which do not require the establishment of vasculature for oxygenation, is not well understood [126]. The mechanism of BM angiogenesis in hematological malignancies is more complex than that of tumor angiogenesis. The vasculature of BM is peculiar and consists of small vessels called sinusoids which have distinctive structural and functional properties that differentiate them from other malignant tissues [53].

There are several methods for evaluation of angiogenesis in AML such as measuring the serum or plasma level of circulating angiogenic factors, evaluation of cellular expression of angiogenic factors in leukemic blasts by flowcytometry [126], quantification of the number of circulating endothelial progenitor cells (cEPCs) by flow cytometry in peripheral blood [127], measuring of bone-marrow microvessel density (MVD) [128]. MVD is done by immunohistochemical analyses on BM and dynamic contrast-enhanced magnetic resonance imaging (DCE-MRI) which is a noninvasive technique that quantifies global and functional BM angiogenesis in situ [129]. Recent research on the vascular system of BM has shown that patients with AML exhibit an elevated amount of MVD and that angiogenesis is closely associated with leukemogenesis. Moreover, patients with high baseline MVD were found to have a poorer prognosis, which aligns with observations in solid tumors [130]. On the other hand, MVD decreased dramatically on day sixteen post-chemotherapy induction in AML patients and it retained its normal level after complete remission (CR) had been achieved [131].

Various cytokines and signaling pathways have been implicated in malignant angiogenesis, including vascular endothelial growth factor (VEGF) and angiopoietins (ANGs). VEGF is secreted by AML cells to activate the VEGF receptor, which can be found in both AML and ECs. VEGF acts on ECs leading to stimulation of various growth factors like CSF3 and IL6 which enhance AML survival, angiogenesis, and proliferation [132]. Additionally, it was been found that VEGF has an anti-apoptotic effect as it activates the *BCL2* family which acts as a shield protecting the leukemic cells from apoptosis [133]. Moreover, elevated plasma levels of VEGF in AML patients have been associated with a reduced survival rate and a lower incidence of achieving CR. Patients with increased VEGF expression also tend to have a shorter disease-free life [134]. Anti-VEGF (aflibercept) showed an increase in the OS in combination with doxorubicin in experimental studies [135]. Moreover, bevacizumab yields a favorable CR rate and duration in adults with AML patients who are resistant to traditional treatment approaches [136].

Another cytokine implicated in angiogenesis is the ANGs (ANG1 and ANG2) system. Patients with AML have higher levels of ANG in their serum and plasma, suggesting a potential role for ANG in leukemogenesis. The TIE2 receptor is activated by ANG1, which triggers the activation of the PI3KCA/AKT survival pathway, leading to the enhancement of EC viability. However, ANG2, an antagonist of ANG1, inhibits TIE2 activation, resulting in unstable vessels that are crucial for the onset of angiogenesis by VEGFA. Balanced and sequential expression of VEGF and ANGs is essential for effective angiogenesis. Additionally, high ANG2 level and ANG2/ANG1 ratio were found to be a predictive index for mortality from septic shock in acute leukemia patients with febrile neutropenia due to disturbance in small vessel permeability [137]. Moreover, AML vasculature shows an enhanced vascular permeability, that causes hindrance in the delivery of chemotherapy and turns the BMM into a haven for drug-resistant cells. Therefore, repairing the impaired vascular permeability can be used as an additional therapeutic technique alongside conventional chemotherapy [138, 139]. Trebananib is a neutralizing peptibody that prevents the interaction of ANGs with the TIE2 receptor [139]. It is highly recommended to use a standard angiogenic profile of each AML patient to determine their prognosis and select the most appropriate course of action. This can be achieved by choosing the right angiogenic therapy that targets multiple angiogenic factors. To facilitate multi-center investigations, a universal technique should be developed and applied for this purpose [126].

## Cellular alternation involved in LSC niche development

### Osteolineage cells

The leukemic endosteal niche promotes deficiency in bone mineralization delaying the maturation process of osteoblasts through several mechanisms. In AML, there is unchecked activation of the RANK/RANKL pathway, which is a crucial signaling pathway in bone remodeling, its activation leads to osteoclastogenesis and increases the survival of osteoclasts. There is a loss of fine-tuning between the bone resorption and formation in AML, the receptor activator of nuclear factor κB (RANK) is a transmembrane protein found on the surface of osteoclasts. On the other hand, the RANKL is a surface membrane protein expressed on the surface of osteoblast cells, stromal cells, and AML blasts [140].

For instance, the BM of mice with AML showed a decrease in osteoblastic cells, which worsened the disease by increasing the number of cancerous cells in circulation, increasing the tumor burden in the spleen and BM, and reducing the survival time of the mice. However, maintaining osteoblastic cells by using a serotonin synthesis inhibitor helped to return the BM to normal, decrease the number of tumors, and increase the survival rate of the mice [141].

Also, it has been demonstrated that AML cells stimulate osteoblastic differentiation while blocking adipogenic differentiation of MSCs by releasing bone morphogenetic protein (BMP) that activates SMAD1/5 signaling on MSCs to enhance osteoblast lineage differentiation. Additionally, osteopontin is an extracellular matrix protein produced that helps in the localization of HSC to the endosteal niche, it has been shown that leukemia decreases the levels of osteocalcin produced by osteoblasts and osteoclasts leading to a decrease in osteoblast and bone loss [142]. Several clinical trials investigated proteasome inhibitors like bortezomib [143], carfilzomib [144], and ixazomib [145] as targets for BM remodeling to promote osteoblast differentiation, suppress osteoclast activity, and induce apoptosis in leukemic cells. Moreover, cabozantinib, a receptor tyrosine kinase inhibitor that can inhibit osteoclast differentiation and resorption, modulates RANKL/osteoprotegerin in osteoblast, found to be well tolerated in AML and effectively inhibits the resistance‐conferring *FLT3*/tyrosine kinase domain/F691 mutation [146].

### MSCs

Despite the fact that MSCs in healthy individuals and AML patients usually express the same phenotypic markers, their functions, subtypes, and molecular profiles can be altered [141]. For example, expression of cell-surface molecules involved in interaction with HSCs is decreased, the number of CD271^+^ MSCs that facilitate LSCs proliferation is increased [147], and they harbor specific chromosomal and genetic aberrations that are different from that present in LSCs mostly in chromosome 1, 7, or, 10 [148]. Additionally, in patients with AML, RNA sequencing of BM MSCs at diagnosis unveiled dysregulation of adhesion molecules, cytokines, and proteoglycans, as well as changes in metabolic pathways, endocytosis, and transcriptional and epigenetic markers. MSCs although show overexpression of JAG1 and SCF down-regulation that decrease the support of normal hematopoiesis of HSCs. Moreover, LSCs reprogramme MSCs to enhance the expression of leukemogenesis-promoting factors such as CXCL12 and JAG1, and lower expression of genes associated with the cell cycle [149]. Notably, MSCs may play a critical role in AML relapse and chemotherapy resistance as they are associated with minimal residual disease persistence through supporting low proliferative but highly resistant LSCs [150]. It was demonstrated that co-culturing stromal cells with AML blasts inhibited both the cell contact-mediated route and the cytotoxic drug-induced apoptosis through MYC-dependent mechanisms, so targeting MYC pathway may serve as a potential tool to decrease drug resistance [151].

### ECs in the leukemic niche

Through a highly regulated cross-talk between ECs and leukemic cells involving autocrine and paracrine stimuli, blast cells can create a unique environment that facilitates their own growth and survival [152]. This process has a profound impact on the pathophysiology of leukemia, as it allows the proliferation of these abnormal cells and the inhibition of normal cell growth. AML blast secretes various cytokines, such as IL1 beta and TNF alpha to enhance their attachment to the endothelium [153]. Several axes have been utilized, namely, CD44/selectin E and VLA4/VCAM1. When these adhesive mechanisms combine, it allows the migration of LSC through the vascular wall, leading to the anchoring of leukemic cells outside the BM. Encouraging studies have shown that antagonizing VCAM1 and selectin E can enhance myeloblast chemosensitivity and mobilization, which undermines their protection in the niche and ultimately reduces their survival [107]. Notably, the cross-talk between LSCs in AML and ECs enhances the process of angiogenesis through the DLL4 pathway [154]. Moreover, VEGF stimulates ECs to secrete CSF2 which stimulates AML cells and proliferation [155].

AML induces dramatic remodeling of BM vasculature through increased microvascular density [156]. Using advanced intravital microscopy two recent researches have demonstrated early, progressive, focal, and spatially non-random patterns of vascular remodeling in murine models of human AML with overall expansion of the endothelial compartment and BM microvascular density specific to engraftment of AML [138, 157]. Moreover, there is an expansion in arteriolar (CD31^+^Sca1^high^) ECs and loss of those corresponding to sinusoids (CD31^+^Sca1^low^). Notably, this aggressive vascular remodeling was associated with disrupted sinusoidal structure, reduced overall vessel diameter, increased vascular permeability, and induction of hypoxic BME [138].

At the molecular level, the analysis of the transcriptome of vascular ECs upon human AML engraftment confirms the toxic phenotype of the ECs. Additionally, gene set enrichment analysis revealed several altered processes directly associated with the abnormal vascular phenotype, including vasculature development, angiogenesis, and response to hypoxia. Of note, there is an extreme upregulation of integrins associated with Fak pathway activation in ECs. Moreover, enhanced expression of *Nox4* in ECs and the increase production of ROS in the BM were observed [138].

### Adipocytes and BMAT remodeling

BMAT has a controversial effect on normal hematopoiesis. On the other hand, the altered BMAT promotes the survival and proliferation of leukemic cells. AML cells introduce a lipolytic effect on BMAT leading to the release of free fatty acids that act as a source of nutrient supplements for leukemic cells [158]. Additionally, the adipogenic space in the BM cavity is decreased to allow uncontrolled expansion of leukemic cells [159]. leukemic cells are highly expressing growth differentiation factor 15 (GDF15) which may enhance the morphological alternation of a large adipocyte to a smaller version [160]. This lipolytic activity of GDF15 is promoted by the high expression of lipolytic genes such as *LIPE* and *PNPLA2* [158].

A study by Tabe et al. [161] revealed that the cross-talk between acute monocytic leukemia cells and adipocytes exerts anti-apoptotic action through an increase in fatty acid β-oxidation (FAO) and upregulation of *PPARG*, *FABP4*, and *BCL2* genes. Targeting BMAT remodeling in AML could serve as a therapeutic strategy for AML. For example, *PPARG* agonists could suppress leukemia proliferation and enhance normal hematopoiesis. Additionally, pharmacological inhibition of FAO could serve as a therapeutic target in AML such as avocatin B [162]. Furthermore, etomoxir, another FAO inhibitor showed an increased sensitivity to Ara-C in resistant AML cells [163].

### Neural cells and nervous system

Several studies elucidated that an altered BMM with SNS denervation enhances leukemic cell proliferation and turns the BMM inhospitable for normal HSCs. This manipulation is done indirectly through disruption of nestin GFP^+^ cell quiescence. Mutant nestin^+^ MSCs reduce the numbers of mature osteoblasts through β2-adrenergic signaling, resulting in a decrease of HSC-retention factors, such as CXCL12, SCF, ANG1, and VCAM1 [164]. It was found that administering a B2-adrenergic agonist led to a decrease in LSCs and increased the lifespan of mice with leukemia [164]. This suggests that changes to the B2-adrenergic pathway may have a role in the progression of leukemia. In conclusion, SNS can serve as a potential therapeutic target in AML.

### Immune cells and LSC niche

It is a well-established fact that patients with hematological malignancies exhibit a remarkable ability of their immune system to identify and eliminate cancerous cells. However, the immune system’s natural restraint is ultimately compromised due to the presence of numerous immunosuppressive mechanisms, leading to widespread immunological dysregulation. This dysregulation culminates in the immune system’s failure to control cancerous cells, allowing them to proliferate and metastasize.

#### Lymphocytes

Within the microenvironment of AML, all subsets of T-cells display dysregulation. It appears that AML may contribute to this dysregulation by affecting the expression of both secreted and cell-bound molecules. Moreover, tumor cell antigen presentation is insufficient on its own to activate the T-cell, the TCR costimulatory pathways play a crucial role in regulating T-cell activation. The CD28 family of receptors is essential for proper T-cell activation that includes several members including CD28, and cytotoxic T-lymphocyte associated protein 4 (CTLA4). The cell surface molecules CD28 provide a positive stimulatory signal on the immune response. Quite the opposite CTLA4 exerts a negative effect on T-cell activation [165]. For example, ipilimumab, the anti-CTLA4 blocking antibody has been shown to promote T-cell responses against AML [166]. Ipilimumab is currently being investigated in a clinical trial for AML in combination with decitabine NCT02890329 [167].

PD1 is a crucial negative regulatory surface molecule that silences the T-cell immune activation. The interactions between PD1 on T-cells and its ligand PDL1 control the induction and maintenance of peripheral T-cell tolerance during normal immune responses through inhibition of the proliferation of T-cells and cytokine production [168]. Up-regulation of PDL1 expression on the surface of tumor cells serves as a mechanism to escape the host immune system. One clinical trial revealed that the blockade of PD1 might have a potential role in the cure of some hematological malignancies [169]. Nivolumab is an immune checkpoint inhibitor that binds to the protein PD1 on the surface of T-cells. In combination with azacytidine, nivolumab appeared to be a safe and effective therapy in patients with AML [170]. Furthermore, pembrolizumab showed efficacy in clinical trials for the treatment of refractory AML patients in combination with cytarabine [171] and decitabine [172].

T-regs are known to suppress effector T-cell activity through several complex mechanisms. To be more specific, the production of immunosuppressive cytokines such as IL10 [173], TGFB [174], and IL35 [175]. In addition, regulatory T-cells express inhibitory molecules like CTLA4 on their cell surface, which can directly interact with APCs and effector T-cells to dampen their activity. Furthermore, T-regs can induce anti-inflammatory biochemical signal pathways in APCs and effector T-cells, leading to the suppression of immune responses. Another important mechanism of regulatory T-cells is the consumption of pro-inflammatory cytokines, particularly IL2, which is essential for the activation and proliferation of effector T-cells. By consuming IL2, T-regs can effectively limit the activity of effector T-cells. Finally, T-regs can directly or indirectly kill effector T-cells and/or APCs, leading to the suppression of immune responses [72]. Studies have identified T-regs as a factor that leukemic cells can use to evade immune surveillance. AML patients have a higher frequency of T-regs in their blood and marrow than healthy individuals. Furthermore, T-regs from AML patients have been found to be more potent in their suppressive abilities compared to healthy controls [176]. One study demonstrated that AML can directly activate T-regs by expressing the T-regs costimulatory ligand ICOSLG and driving certain CD4^+^ cells to become inducible T-regs. Moreover, ICOS^+^ T-cells have the ability to create IL10, which has been demonstrated to have an inhibitory effect on T effector cells and promote the growth of AML in turn [177].

It was revealed that CD8^+^ T-cells in the BM of AML patients were more exhausted and had reduced function [178]. It also showed that the expression of T-cell immunoglobulin mucin3 (TIM3), a marker of T-cell exhaustion, and its partner LGALS9, were significantly higher in refractory AML patients [179]. AML cells express a high level of arginase II enzyme that acts by lowering the arginine level leading to T-cell exhaustion [180]. Additionally, some studies showed that there is a definite dysregulation in the Th1/Th2 axis, some studies revealed that, as compared to healthy controls, AML patients had more Th17 cells in their BM and peripheral blood, and more increase in the cytokines produced from these cells [181, 182].

NK-cells, a highly effective subset of cytotoxic lymphocytes, exhibit a potent and versatile immune response against tumor cells, including leukemic cells [183]. AML cells act through the release of immunosuppressive cytokines and the enhanced expression of inhibitory molecules on their membrane. These work together to create an immunosuppressive environment that evades detection by NK-cells and allows the leukemic cells to escape immune surveillance. Additionally, the AML immunosuppressive microenvironment acts by decreasing NK maturation, inhibiting their immune checkpoints, enhancing NK-cell anergy, and alternating the NK receptor repertoire [184].

#### Macrophages

Monocyte-derived macrophages in the BM have shown a unique capability to adapt to the environment maintain homeostasis and respond to inflammation and infection. In solid tumors, neoplastic cells recruit those macrophages, inducing alternation in the stromal cells to allow their differentiation into tumor-associated macrophages (TAMs), In the AML scenario, these cells are named leukemia-associated macrophages (LAMs). Some studies suggest that those LAMs help in chemotherapeutic resistance to cytarabine by inhibition of chemotherapy-induced apoptosis [185].

## Exosomes and alternation in bone microenvironment in AML

Exosomes are tiny vesicles with a size range of 30 to 200 nm, and they are secreted by both normal and malignant cells. These vesicles are emerging as crucial players in facilitating communication between cells both on short and long distances. Their importance in cellular communication is increasingly being recognized [186]. Exosomes are one of the subtypes of extracellular vesicles that can act as a vehicle for the transfer of signaling molecules, as well as microRNAs (miRNAs) and functionally active genes [187]. Additionally, it was found that exosomes are the most important subtype of extracellular vesicles (EVs) as they carry a potential reservoir of disease biomarkers and may serve as a therapeutic target.

One study supported that AML cells definitively alter the BM niche by secreting exosomes, which strongly promote leukemic cell survival while simultaneously inhibiting normal hematopoiesis, and also showed that plasma exosomes were significantly increased in AML patients compared with healthy controls [23]. Moreover, the plasma level of exosomes is altered according to response to chemotherapy [188]. Plasma-derived exosomes in AML were found to contain not only standard exosome markers like tetraspanins but also membrane-associated TGFB1, and myeloid blast markers CD34, CD33, and CD117. This unequivocally indicates that the source of these exosomes was leukemic blasts [189, 190]. AML exosomes were found to be enriched in coding and non-coding RNAs related to creating and maintaining the leukemic niche formation (IGFIR, CXCR4, MMP9). Notably, AML exosomes miRNA can be transferred to bone marrow stromal cell (BMSC) leading to a significant alteration in their functions [191]. Leukemic cells produce exosomes that have the ability to modify the functions of various tissue cells, particularly EC, resulting in pro-angiogenic properties. Exosomes can facilitate the remodeling of EC and stimulate the formation of angiotubes by moving freely within and between the nanotubular structures that connect them. The exosomes cause the surface of EC to lose B-catenin and VE-cadherin, which in turn increases EC mobility [192].

Furthermore, leukemic exosomes can affect the immune cells in the BM to support the leukemic niche development. For example, NK-cell activity was found to be downregulated by exosomes carrying TGFB1 in AML plasma. NK-cell cytotoxicity was restored with anti-TGFB1 specific antibodies which can also suppress T-regs [189]. The studies leave no doubt about the crucial role that leukemia-derived exosomes play in supporting leukemic cells within the BMM. Additionally, the exosomes secreted by the BMM tend to favor leukemogenesis as well.

## Conclusions

Each normal and leukemic BM niche possesses a unique microenvironment and molecules. These niches have their own stem cells, as well as signaling pathways, microenvironment-specific chemokines, and chemokine receptors that are crucial in the development and survival of cancerous and normal stem cells. LSCs have an instructive role in the BM microenvironment that can contribute to the generation of a supportive niche for leukemic cells themselves. Modern techniques like scRNA-seq and high-resolution microscopy have the potential to uncover a wealth of information about the BMM, cellular interactions, and genetic expression within the niche of myeloid malignancies. By leveraging these methods, researchers can gain deeper insights into the complex pathways involved in the development and progression of these malignancies. This comprehensive understanding of similarities and differences between normal and malignant BM niches and LSCs heterogeneity can pave the way for the identification of novel therapeutic targets and the development of more effective combination therapeutic strategies to target the LSCs and their malignant niche, eliminate minimal residual disease, and drug resistance mechanisms. This approach may lead to a decrease in the incidence of relapse and an increase in the OS in AML. In our opinion, functional assessment of niche contribution to AML and pre-clinical testing of new niche-targeted therapies require the establishment of disease-relevant model systems. Researchers’ efforts should be focused on building bone/BM organoids systems that aim to closely recapitulate the human malignant BM niche complexity.

## Abbreviations

AML: acute myeloid leukemia
ANGs: angiopoietins
APCs: antigen presenting cells
BM: bone marrow
BMAT: bone marrow adipose tissue
BMM: bone marrow microenvironment
CAR: CXCL12-abundant reticular
CR: complete remission
CSF3: colony-stimulating factor 3
CTLA4: cytotoxic T-lymphocyte associated protein 4
EC: endothelial cells
EH: extramedullary hematopoiesis
FAO: fatty acid β-oxidation
HSCs: hematopoietic stem cells
IL1: interleukin 1
LSCs: leukemic stem cells
MRD: minimal residual disease
MSCs: mesenchymal stromal cells
MVD: microvessel density
NK: natural killer
OS: overall survival
PD1: programmed death 1
PDL1: programmed death ligand 1
RANK: receptor activator of the nuclear factor κB
RANKL: receptor activator of the nuclear factor κB ligand
SCF: stem cell factor
scRNA-seq: single-cell RNA sequencing
SNS: sympathetic nervous system
TGFB: transforming growth factor-beta
T-regs: regulatory T-cells
VCAM1: vascular adhesion molecule 1
VEGF: vascular endothelial growth factor
VLA4: very late antigen 4

## Declarations

### Copyright

© The Author(s) 2024.
